# High-resolution multilocus sequence typing for *Chlamydia trachomatis*: improved results for clinical samples with low amounts of *C. trachomatis* DNA

**DOI:** 10.1186/s12866-020-02077-y

**Published:** 2021-01-18

**Authors:** Shlomo Pilo, Gal Zizelski Valenci, Mor Rubinstein, Lea Pichadze, Yael Scharf, Zeev Dveyrin, Efrat Rorman, Israel Nissan

**Affiliations:** Ministry of Health, National Public Health Laboratory, Tel Aviv, Israel

**Keywords:** *Chlamydia trachomatis*, Multilocus, Sequence, Typing, MLST, Melting curve analysis, Genovar

## Abstract

**Background:**

Several Multilocus Sequence Typing (MLST) schemes have been developed for *Chlamydia trachomatis*. Bom’s MLST scheme for MLST is based on nested PCR amplification and sequencing of five hypervariable genes and *ompA*. In contrast to other Chlamydia MLST schemes, Bom’s MLST scheme gives higher resolution and phylogenetic trees that are comparable to those from whole genome sequencing. However, poor results have been obtained with Bom’s MLST scheme in clinical samples with low concentrations of Chlamydia DNA.

**Results:**

In this work, we present an improved version of the scheme that is based on the same genes and MLST database as Bom’s MLST scheme, but with newly designed primers for nested-1 and nested-2 steps under stringent conditions. Furthermore, we introduce a third primer set for the sequencing step, which considerably improves the performance of the assay. The improved primers were tested *in-silico* using a dataset of 141 Whole Genome Sequences (WGS) and in a comparative analysis of 32 clinical samples. Based on cycle threshold and melting curve analysis values obtained during Real-Time PCR of nested-1 & 2 steps, we developed a simple scoring scheme and flow chart that allow identification of reaction inhibitors as well as to predict with high accuracy amplification success. The *improved MLST* version was used to obtain a genovars distribution in patients attending an STI clinic in Tel Aviv.

**Conclusions:**

The newly developed MLST version showed great improvement of assay results for samples with very low concentrations of *Chlamydia* DNA. A similar concept could be applicable to other MLST schemes.

**Supplementary Information:**

The online version contains supplementary material available at 10.1186/s12866-020-02077-y.

## Background

*Chlamydia trachomatis* is a Gram-negative, obligate intracellular bacterium, responsible for a wide range of diseases [1]. Although *C. trachomatis* infections are often asymptomatic, late complications increase the risk of ectopic pregnancy and infertility when untreated [2, 3]. In addition to being the most prevalent sexually transmitted bacteria worldwide, with an estimated annual 131 million new cases [4], *C. trachomatis* infections are associated with cervical cancer, and facilitate the transmission of HIV [5–8]. *C. trachomatis* DNA is comprised of a ~ 1 million base pairs (bp) long single circular chromosome, as well as multiple copies of a 7.5 kb long highly conserved plasmid [9–11]. Based on the antigenic properties of the major outer membrane protein (MOMP), *C. trachomatis* is typically divided into 17 distinct serovars [12–14], or 19 serovars which include subtypes [15]. The serovars include: trachoma serovars (A-C) that are the major etiological agents of preventable blindness; genital tract sexually transmitted serovars (D-K); and serovars L1–L3 that cause invasive urogenital infection or anorectal infection (lymphogranuloma venereum, LGV). Characterized by ulcerative proctitis [16], the LGV disease is of high concern [12–15, 17–19].

Since the advent of sequencing, sequence analysis of the MOMP gene (*ompA*), which encoded by nearly 1200 bp, has been widely used for epidemiological studies [20]. Genotypic variation within the *ompA* gene exhibits a great degree of polymorphism that cannot be identified by serotyping [21–23]. However, *ompA* variability does not provide sufficient discriminatory power for epidemiological purposes [12, 24]. Although *C. trachomatis* genome harbor regions with highly nucleotide diversity and high events of recombination, it is considerably conserved [25, 26]. The genome is characterized by a low level of genetic diversity among variants (< 2% of the genome), and the *ompA* genotype classification strongly correlates with tissue tropism and disease outcome [26, 27].

Adding to the challenges described above, the *C. trachomatis* pathogen has an obligate intracellular life cycle. Clinical DNA samples contain only a small quantity of *C. trachomatis* residues mixed with DNA of both human cells and numerous diverse microorganisms. Hence, in order to obtain sufficient amounts of genomic DNA, for molecular typing, inoculation of clinical specimens and subsequently generating sufficient amounts of genomic DNA, requires an extensive in vitro culturing process, which may lead to genomic changes due, for example, to the absence of host immune pressures [12, 28–31].

In order to overcome the low discrimination power of the above-mentioned techniques, several genotyping systems have been developed [30]. Two groups developed multilocus sequence typing, based on the sequences of seven housekeeping genes (MLST-7) of *C. trachomatis*; Pannekoek el 2008 [32] and Dean et al. 2009 [33]. both schemes are supported by the PubMLST [34]. The MLST-7 method has been useful for exploring long-term and global epidemiological trends [13, 32, 35]. In order to explore partner tracing and molecular epidemiology of short-term outbreaks, a second multilocus sequence typing was developed, MLST-5 [36]. It is based on the investigation of five target variable regions of the Chlamydial genome: *hctB*, CT058, CT144, CT172 and *pbpB*. Although this method achieved high resolution it is not optimal for direct amplification from clinical samples. Therefore, the scheme was later improved by Bom et al. [28]. Bom’s study used regions up to nearly 700 bp in length for five target regions (the sixth target region is ompA ~ 800 bp), improving PCR sensitivity, reducing PCR targets, and making it easier to assemble [28]. In a later study, additional advances were made in the method by redesigning two of the primers and adding M13-tailed primers [8]. The final protocol for primers and cycling conditions is described on the pubMLST website (http://mlstdb.bmc.uu.se/current.html), [34].

Despite these improvements clinical samples containing small quantities of Chlamydia DNA, as determined by GeneXpert (Xpert® CT/NG), did not show sufficient performance. Therefore, the aim of this study was to design novel primers for nested-1 and nested-2 PCR, as well as a third primer set for the sequencing step. In addition, we developed a simple scoring scheme, based on the Real-Time PCR assay cycle threshold (CT) value and melting curve analysis, which predicted amplification success with high accuracy. Our method improved MLST results for clinical samples with very low amounts of *C. trachomatis* DNA. The methodology used during this study can be applied to other MLST schemes.

## Results

**Fig. 1:**
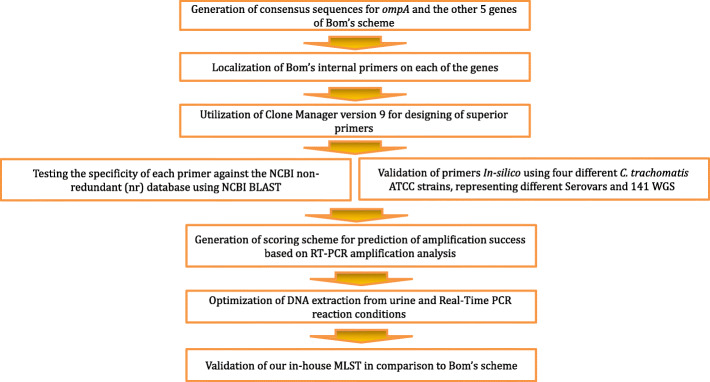
Flowchart of the strategy that we utilized in our MLST primer designing

The strategy that we used in our MLST primer designing is drawn in Fig. 1. In general, we constructed a consensus sequences of *hctB* (CT046), CT058, CT144, CT172, *pbpB* (CT682) and *ompA* by running Clustal W with BioEdit 7.2.5 on datasets representing major and diverse genovars. On each consensus sequence, we localized the internal primer of Bom’s [28] in order to use them as a flag. Our novel primers were located according to this flag and the specificity of each primer against the NCBI non-redundant (nr) database and Human RefSEqGene Sequences (RefSeq_Gene) using NCBI BLAST were tested. Accepted criteria for primer were as followed: identification of various serovars of *C. trachomatis*, and absence of human sequences. The primer list appears in Table 1 and the consensus gene and primer location in the supplementary information Figures S1, S2, S3, S4, S5 and S6. Using stringent criteria during primer designing, we selected primers that gave significantly better scoring by Clone Manager 9.0 (Sci-Ed Software) (supplementary Tables S1, S2). As a result, the average deviation per primer from the design criteria (as calculated by dividing the total number of deviation from the design criteria by the numbers of primers), in our scheme is 0.28 versus 2.13 in Bom’s MLST scheme [28]. Comparative data about primers of Bom’s MLST scheme and primers presented in this work appears in the supplementary information Tables S1, S2, S3, S4, S5 and S6.

**Table 1:**
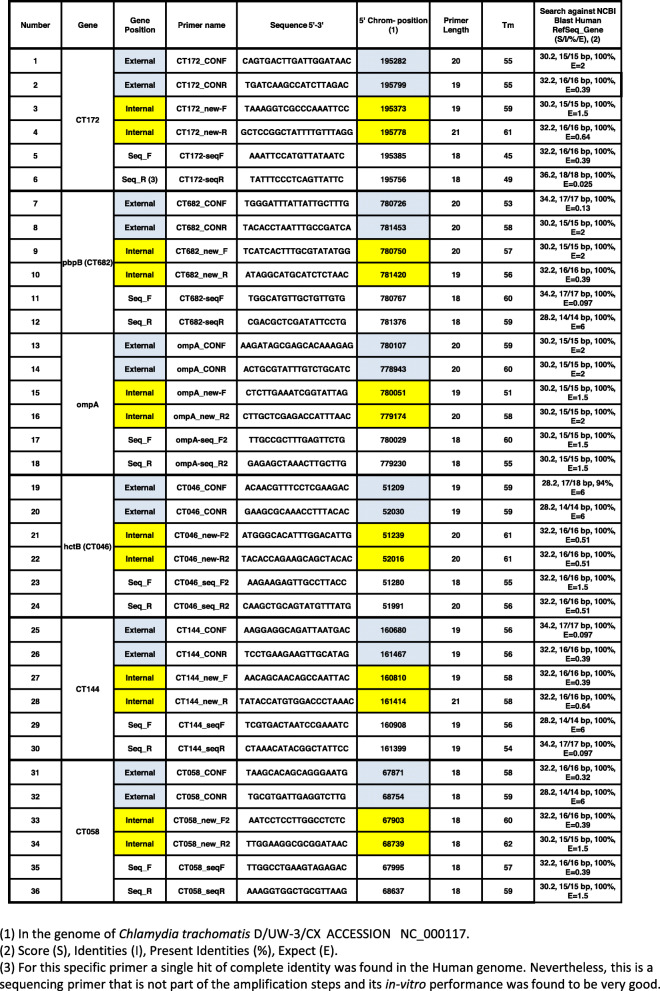
MLST PCRs newly designed primer list. The colors signify the different groups of primers: light blue, external gene position (Nested-1). Yellow, internal gene position (Nested-2). White, primers used for sequencing

*In-silico* analysis was performed in order to ensure the ability of the primers to amplify genes from a wide range of natural isolates, represented by 141 high quality genome sequences of *C. trachomatis* from the NCBI Genome Sequences database. Using R script we calculated the binding and amplification potential of Bom’s primers versus our newly designed primers. The analysis showed that the primer pairs were able to bind to the correct target, in a proper orientation and distance to allow efficient PCR amplification. The *in-silico* analysis confirmed that our primers were able to bind to a high proportion of the sequences with a very low number of mismatches (not more than two mismatches for a primer, supplementary information, Table S3, S4, S5 and S6).

**Fig. 2:**
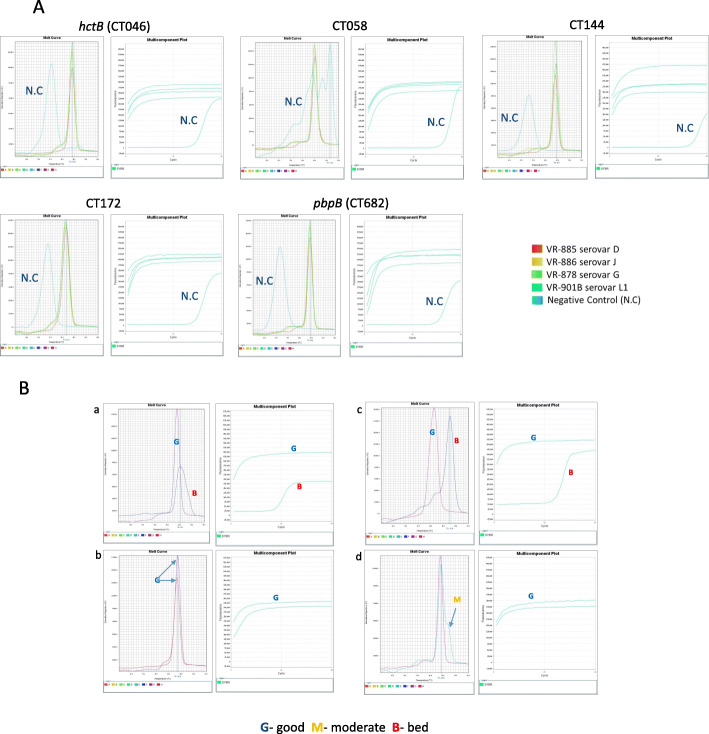
Representative results for typical nested 2 step amplification and melting curve analysis for each of the *hctB* (CT046), CT058, CT144, CT172 and *pbpB* (CT682) genes, the left figure shows the melting curve and the right the amplification plot (multicomponent plot). A) typical successful nested 2 step PCR amplification of the ATCC strains: ATCC VR-885, ATCC VR-886, ATCC VR-878 and ATCC VR-901B. B) Typical successful and unsuccessful nested 2 step PCR amplification of clinical samples: G (Good) successful amplification, B (Bad) Unsuccessful amplification and M (Moderate). Unsuccessful amplification of CT058 of sample 298 versus positive control (a), Successful amplification of CT046 of sample 293 versus positive control (b), Unsuccessful amplification of CT172 of sample 230 versus positive control (c), and Moderate amplification of CT682 of sample 362 versus positive control (d)

To test our new primers for their ability to accomplish successful MLST, we selected four different ATCC strains of *C. trachomatis* representing different serovars ATCC VR-885 (D), ATCC VR-886 (J), ATCC VR-878 (G) and ATCC VR-901B (L1). Amplification and sequencing was carried out as described in the material and methods section. We noticed that regardless of the serovar, all these strains gave almost identical melting curves for the five genes of the MLST and *ompA* (Fig. 2A). We utilized this finding for the development of a scoring scheme for amplification success (Fig. 2B, next paragraph). To validate our MLST results, we downloaded the *C. trachomatis* ATCC strains sequences from the NCBI nucleotide gene bank (when available), and interrogated them for the alleles in the Uppsala MLST database (http://mlstdb.bmc.uu.se/current.html). The *ompA* genovar was confirmed by blastn search. The results were compared to STs and *ompA* genovars obtained by in-vitro amplification and sequencing using the same strains DNA. Identical results were obtained (Table 2). The ability of our method to achieve identical results to the Bom’s MLST scheme [28] was further confirmed in a comparative study of 32 clinical samples (Table 3, supplementary information Table S8). Each sample was tested once. We used identical reaction condition between the two methods (Methods), except the specific primers, in order to be focused only in the primers contribution to the success rate of the method. The fit between the two methods was found to be excellent (97.7% agreement in allele identification, 100% agreement in genovar identification). The newly designed method showed the best scores as shown in Table 3. The Limits of Detection (LOD) was determined to be 1–10 copies of chromosomal DNA using three *C. trachomatis* ATCC strains (Table 4).

**Table 2:**
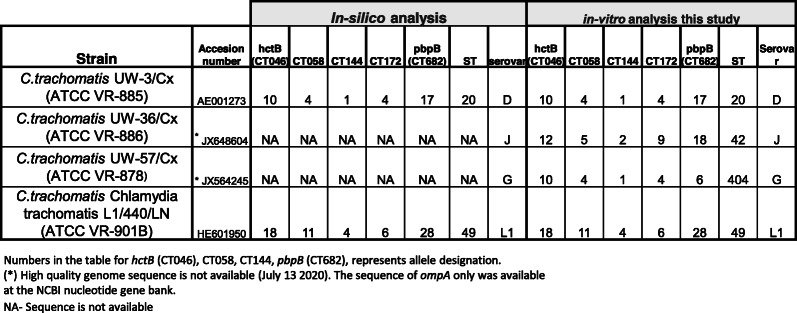
Sequencing of *C. trachomatis* ATCC strains. Sequences were downloaded from the NCBI nucleotide gene bank (when available) or determined de novo using our in-house MLST scheme. The alleles were determine by Uppsala MLST database interrogation and the *ompA* genovar was determined by Blast search against the nr NCBI database. In all cases, the results of the de novo sequencing were identical to the sequence downloaded from the NCBI database

**Table 3:**
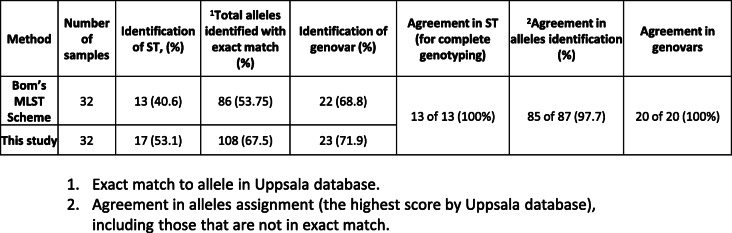
Comparative results of Bom’s MLST scheme [28], and this study on 32 positive clinical samples that were obtained from a community clinic for STI’s and HIV in Tel-Aviv. The total number of alleles (except *ompA*) is 160 (=32X5)

**Table 4:**
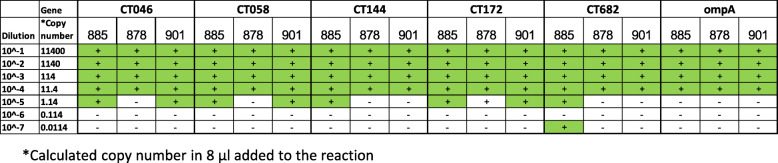
Limits of Detection (LOD) for each of the *hctB* (CT046), CT058, CT144, CT172, *pbpB* (CT682) and *ompA* genes, using the new primers schemes, of the ATCC strains: VR-885, VR-878 and VR-901B. + Positive detection. – Negative detection. The LOD is between 1 and 10 genomic copies (green)

**Fig. 3:**
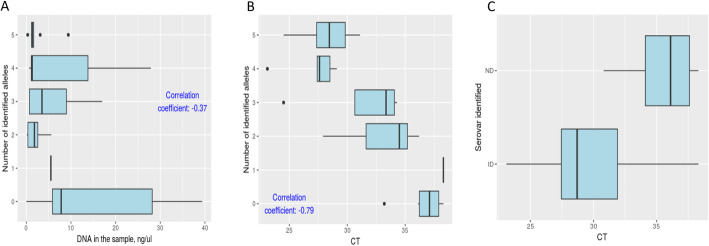
The correlation between DNA concentration (**a**) and threshold cycle (**b**, **c**) in the sample to sequencing successes are shown in the boxplots. **c** Correlation between threshold cycle to sequencing successes of *ompA*. Odds of success are high when CT≤33. Above this value, success rates decrease

We successfully developed scoring scheme for the prediction of amplification successes (material and methods and Fig. 2B). In 96.9% of 522 reactions, good agreement was found between the amplification qualities to the sequencing qualities (supplementary information Table S7). Notably, in only 3.1% of the samples no agreement was found, confirming that our scoring scheme can be used reliably. In general, this methodology can be applied to any MLST scheme. No significant correlation was found between the total nucleic acid concentrations in the sample to the sequencing success, representing the great challenge of obtaining Chlamydia sequences from a highly complex natural sample (Fig. 3a). Odds of success are high when CT≤33 (89.5%). Above this value, success rates decrease (Fig. 3b, c).

Based on the amplification score we developed a useful flow chart (supplementary information Figure S7), that allowed us to identify problems during the MLST (like the presence of reaction inhibitors, poor or no amplification, etc.) and to take corrective steps in order to improve the MLST success rate.

**Fig. 4:**
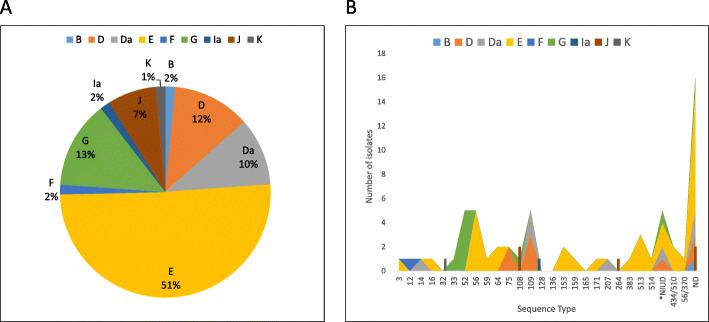
Genovars and MLST distribution of the 81 clinical samples from patients attending community clinic for STI’s and HIV in Tel-Aviv. **a** Serovar of 67 out of 81 urine samples (82%). **b** Integration of the genovars and MLST distribution of 52 out of 81 samples (64.2%). NIUD – Not In Uppsala Database, represent novel ST’s. ND- Not Determined. ST\ST - there are two options for sequence type (434\510, 56\370)

We applied our MLST scheme on 81 Chlamydia positive clinical urine samples as tested by GeneXpert collected during the years 2015–2016 from patients attending a community clinic for STI’s and HIV in Tel-Aviv (supplementary information Table S9). The genovars distribution and the correlation between genovar and sequence types (ST) of the clinical samples appear in Fig. 4a and b respectively. The patients attending this clinic were not required to identify themselves. The provision of personal information, including sexual preference, sex, age, etc., had permission solicited but the details were not verified. We were able to obtain *ompA* sequences from 67 out of 81 urine samples (82%) and complete MLST data (five genes) for 52 out of 81 samples (64.2%). Interestingly 5 samples included alleles combination that did not appear in the Uppsala database therefore represents novel STs. For example, sample number 296 contains the following alleles combination: hctB-10, CT058–6, CT144–7, CT172–2, CT682–1. The appearance of hctB-10, CT058–6 in the combination with the other alleles is novel. The three most prevalent genotypes were E (*n*=34; 51%), G (*n*=9; 13%), and D (*n*=8; 12%). The STs of the samples were highly diverse with ST52, 56 & 109 appearing at higher numbers (Fig.4b). LGV genotypes were not detected in this samples set.

The amount of DNA template in the nested-1 step was very important and we found that we can obtain good sequences in several cases of failure, by increasing the volume of nested-1 to 40 μl thus doubling the volume of template DNA (from 8 μl to 16 μl) (not presented).

## Discussion

The aim of this study was to design a new nested PCR MLST scheme based on Bom’s MLST scheme [28], making it more suitable for clinical samples containing a large proportion of irrelevant DNA (from human and diverse microorganisms) with only a small amount of Chlamydia DNA. To achieve this goal, we redesigned nested-1 and nested-2 PCR primers using highly stringent criteria, and introduced a third primer set for the sequencing step. Using clinical urine samples, our version identified the same alleles as the original Bom’s MLST scheme [28] in almost all of the cases (97.7%), while allowing identification of a higher number of alleles (13.8% increase), Table 3. Nevertheless, comparative analysis of more and divers samples (from different body sites, paper in preparation) are needed in order to support the superiority of our method.

This study showed that careful planning of nested-1 and nested-2 and sequencing primers and the utilization of various *in-silico* analyses, enabled improvement in amplification and sequencing success rate. In a typical thermocycler, the success or failure of reaction cannot be predicated and is apparent only after accepting the sequence. Therefore, the ability to apply changes or corrections is very limited. On the contrary, our method allow efficient real-time intervention as needed in order the retrieve the maximal information from the sample. By using this method during the procedure, the user can take a calculated decision (Figure S7). The development of a scoring scheme and flow chart to assess amplification success allowed us to overcome cases of reaction inhibitors, poor or no amplification and to improve the MLST performance. In fact, a similar scoring scheme can be applied to any MLST scheme. Note that no high resolution melting analysis (HRMA) was used nor melting curve results, for purposes of genotyping as described by Giffard PM et al., 2018 regarding *Ct*GEM typing method [37].

Our MLST scheme was used successfully for analysis of various clinical samples including vaginal and rectal swabs (not shown). By adjusting the amount of nucleic acid taken for nested PCR-1, as described in the material and methods section, most of the PCR inhibition was prevented. This study presents for the first time data about Israel genovars and ST’s distribution among *C. trachomatis*. Notably, the most prevalent genotype of isolates from patients attending an STI clinic in Tel Aviv is genotype E, which accounts for about half of the isolates. Interestingly, the genovar’s distributions are similar to the report from European countries [24, 27] and Tunisia [38], but present differences from Asian countries such as China, where the genotypes D and G were the most common [39], and India where genotype D was found to be most prevalent [40].

In the paper of Björn Herrmann et al., (2015) he describe the genovars and STs distribution among 2089 specimens from 16 countries [24]. In that work they found that the most common STs among heterosexuals are 3, 12, 55 & 56 and in men having sex with men (MSM) the predominating STs are 52, 58, 108 & 109. Our data shows that the predominate STs in Israel are 52, 56 and 109 and this is the first report from Israel. Our data expand the knowledge about the global distribution of these strains.

The novel MLST scheme presented in this paper was adopted as the official working procedure of the Chlamydia Reference Laboratory in the Israeli National Public Health Laboratories, Tel-Aviv.

### Study limitations

For the comparative analysis, we used 32 samples (from total of the 81 samples). This is a relatively small number. In the real world, the performance of the assay might be affected by sampling, transport condition and other unknown factors. Theoretically, in other sets of samples the success rate might be different. Analyzing more samples from diverse sources (not just urine) will allow us to test the robustness of this new method in comparison to other MLST schemes. Nevertheless, *in-silico* test of our primers on a wide range of *C. trachomatis* whole genomes demonstrated the primers potential for sequencing a wide variety of *C. trachomatis* natural isolates.

Additional limitation was the use of different reaction volumes from the Bom’s MLST scheme [28]. While we used reaction volumes of 20 μl and 10 μl for nested-1 and neasted-2 respectively, Bom’s MLST method presents 25 μl reaction volumes for both nested-1 and nested-2. As indicated in the material and method and in the results sections, we used identical reaction condition for both methods, except the primers, in order to focus the study in the primers contribution to the success rate of the process.

## Conclusions

Taken together, *in-silico* and in-vitro comparison of our redesigned primers verses Bom et al. 2011 primers [28], found that our novel primers performed better. Notably, Bom et al. 2013 published a redesign of the pBpB 2366 R and CT 1678 R primers in order to improve their specificity [8]. This emphasizes the importance of careful primers designing. In conclusion: Our method introduces many advantages to the Bom’s MLST scheme. Many of the principles developed during this study can be easily adjusted to other MLST schemes, such as Chlamydia MLST-7 or any other bacterium’s MLST determination.

## Methods

### DNA and clinical samples

DNA of the following strains were purchased from the ATCC (https://www.atcc.org/): ATCC VR-885 (D), ATCC VR-886 (J), ATCC VR-878 (G) and ATCC VR-901BD (LGV1).

81 *C. trachomatis* positive urine samples, collected during the years 2015–16 were stored at − 80 °C until DNA extraction.

All samples were examined by GeneXpert (Cepheid, Xpert® CT/NG) with a reagent kit for detection and differentiation of *Chlamydia trachomatis* and *Neisseria gonorrhoeae* (catalog number GXCT/NG-CE-10), used according to the instruction of the manufacturer.

### Primer design

The general scheme is described in Fig. 1. In details, for each target gene DNA sequences were downloaded from NCBI, the DNA representing various genovars (urogenital, LGV). A consensus sequence was created using Clustal W (BioEdit ver 7.2.5). The consensus sequences were uploaded into Clone Manager 9.0 (Sci-Ed Software) and the internal primers of Bom’s [28] scheme were located on the consensus in order to serve as a flag for localization of the new primers. We used stringent criteria (supplementary information, Tables S1, S2) to design new primers. Tables S1, S2 include detailed information on each primer. The consensus sequences for the MLST-5 and *ompA*, including the position of the primers appear in the supplementary information Figure S1, S2, S3, S4, S5 and S6. The specificity of each primer was further tested by running blastn against the NCBI Human RefSeqGene Sequences (RefSeq_Gene) database and by *in-silico* amplification analysis against 141 *C. trachomatis* WGS (supplementary information, Tables S3, S4, S5 and S6).

### DNA extraction

Nucleic acids were extracted from 1.5 ml of Chlamydia-positive urine samples, using the MagNA pure compact Instrument (Roche), according to the manufacturer’s instructions. In short, frozen urine samples were left to thaw at room temperature. Thawed samples were centrifuged for 10 min at20,000 g. At the end of the centrifugation most of the supernatant was removed keeping 200 μl for resuspension of the pellets. 200 μl of MagNa Pure Lysis Buffer (catalog number 04659180001) and 20 μl of Proteinase K (Roche, 04909640001) were added and the samples were incubated at 65 °C and 95 °C for 10 min each. After lysis the lysate was left to cool down at room temperature for 5 min. 400 μl of lysate were loaded into the MagNA Pure Compact Instrument (Roche) and the nucleic acids were extracted using MagNA Pure Compact Nucleic Acid Isolation Kit I (Roche, catalog number 03730964001). The nucleic acids were eluted in 100 μl.

### Measurement of the DNA concentration

The concentrations of the nucleic acids extracted from clinical samples were measured using nanodrop 2000. Magnetic beads purified DNAs after the nested-2 step PCR (as described at the “Cleaning PCR products” section) were measured using the DeNovix ds broad range kit (catalog KIT-DSDNA-BROASD-2) with DeNovix QFX Fluorometer or by the PicoGreen (Invitrogen, P11496) method using TECAN SPECTRAFOUR Plus, according to manufacturer’s instructions. The nucleic acids DNA concentration in the sample was used to adjust the amount taken for the PCR reaction. Samples with up to 10 ng/μl were used as is; for 11–100 ng/μl the sample was diluted 1 volume sample plus 9 volumes PCR GRADE WATER (HyLabs, Cat number BP556/100S(, before taken to for PCR; for over 101 ng/μl the sample was diluted 1 volume sample plus 99 volume PCR GRADE WATER before taken for PCR. These adjustments helped overcome cases of PCR inhibition, mainly in vaginal swabs that gave extremely high yields of nucleic acids.

### Nested PCR for chlamydia trachomatis

The method is based on two consecutive PCR reactions and sequencing of the MLST regions. In the first PCR (nested-1) we used external primers and in the second PCR (nested-2) we used more internally located primers. For sequencing we used a third set of primer that is located even more internally and was not involved in the PCR amplification step (Table 1, see supplementary information, Figures S1, S2, S3, S4, S5 and S6 for sequence & graphical presentation). StepOnePlus Real-Time PCR (Applied Biosystems, Waltham, MA, USA) was used for PCR amplification during this study. The amplification of each target gene was performed separately.

### Nested-1 PCR

In the first PCR reaction, we used sets of external primers (HyLabs Israel LTD, Table 1) for DNA amplification on the regions of *hctB (CT046), CT058, CT144, CT172, pbpB (CT682) and ompA*. The amplification was performed in a volume of 20 μl containing 8 μl of extracted DNA, 0.02 U/μl KOD Hot-Start DNA polymerase (EMD Millipore Corp., 71,086–3), 1.5 mM MgSO_4_, 0.2 mM each deoxynucleoside triphosphate (dNTPs) and 0.5 μM of each specific outer primer. The fluorescent dye LightCycler® 480 ResoLight Dye (Roche, 0490964001) was used in the reaction at final dilution X40. Cycling conditions were 120 s polymerase activation step at 95 °C, followed by 45 cycles of denaturation at 95 °C for 20 s, annealing at 53 °C for 15 s and extension at 70 °C for 20 s for 500-1000 bp target. Melt curve: 95 °C for 15 s, 60 °C for 60 s and up to 95 °C, measuring light emission every 0.3 s. *C. trachomatis* ATCC strain DNA diluted from 1:500 to 1:2000 was used as a positive DNA control.

### Nested-2 PCR

The consecutive reaction was performed using 1 μl of nested-1 reaction in a total volume of 10 μl. The reaction contained internal primers as described in Table 1, 0.02 U/μl KOD Hot-Start DNA polymerase, 1.5 mM MgSO_4_, 0.2 μM each deoxynucleoside triphosphate and 0.5 μM of each specific outer primer, LightCycler® 480 ResoLight Dye at X40 final dilution. Cycling conditions were 120 s of polymerase activation step at 95 °C, followed by 40 cycles of denaturation at 95 °C for 20 s, annealing 51 °C for 15 s and extension at 70 °C for 20 s. Melting curve analysis was performed as in nested-1.

### Cleaning PCR products

At the end of nested-2 PCR reactions, the PCR reactions were cleaned by the Agencourt AMPure® XP® (Beckman Coulter, A63881) PCR purification system of paramagnetic bead technology. For each of the 10 μL PCR reaction, 18 μL of rigorously re-suspended beads were added. We separated the DNA from the beads using the Agencourt SPRIPlate Super Magnet Plate (Beckman Coulter A32782), followed by two washes with 100 μL of 70% ethanol, and elution with 40 μl of 1xTE solution (Tris-EDTA buffer, 100x Concentrate, Sigma Aldrich, Cat # T9285-100ML). The purified DNA was stored in − 20 °C until use.

### Bom’s MLST scheme

The protocol is based on the primers described in Bom RJ et all., 2011 [28] with the use of the reaction condition exactly as described for our method.

### Sequencing analysis

The amount of the purified DNA was measured using the PicoGreen or DeNovix assay as indicated and the DNA was diluted according to the HyLabs Israel LTD instructions prior sequencing. Each individual amplification product was cleaned and was sequenced from both sides, using the internal Seq_F and Seq_R primers (Table 1). Next, the sequences were examined with the Chromas ver. 2.6.5 (Technelysium DNA sequencing software) and DNA sequencing quality was inspected. Gene contigs were generated by uploading the forward and reverse sequences of each gene into Clone Manager 9.0 (Sci-Ed Software). Cases of disagreements between the forward and reverse sequences were resolved by using the best chromatogram. The assembled sequences were saved in a FASTA format. In order to get MLST Sequence Type (ST), we used the Uppsala, Sweden, *C. trachomatis* MLST database website (http://mlstdb.bmc.uu.se/current.html) [34] for sequences interrogation. For *ompA* genotyping we used Blastn at the NCBI database (https://blast.ncbi.nlm.nih.gov/Blast.cgi).

### Developing of scoring system for prediction of PCR amplifications success

We utilized the Real Time PCR Melt Curve (MC) and cycle threshold (CT) diagrams (obtained from the nested-2 PCR step) in order to predict amplification success. The melting curves were compared to those of the positive control. The amplifications were classified into three categories:

- Good (G): CT ≤ 20, melting curve with a single peak with Tm within 1 °C from the Tm of the positive control,
- Bad (B): CT > 20 and a single peak more than 3 °C from the Tm of the positive control or multiple peaks,
- Moderate (M): CT ≤ 20 and a single dominant peak 1 °C − 3 °C from the Tm of the positive control and possibly a secondary non-specific peak or, CT > 20, melting curve with a single peak with Tm within 1 °C from the Tm of the positive control (Fig. 2). For example, if the MC is identical to the positive control and the CT≤20 in nested 2 step, the score will be Good (G), but if the MC represents multiple peaks, or a major peak with a Tm that is significantly different from the positive control (more than 3 °C), the score will be Bad (B). For more examples, see Fig. 2B. The correlation between the amplification score (G/M/B) to sequencing successes was determined.

### Limits of detection (LOD)

We used ATCC certificate of analysis data of the following strains ATCC VR-885 (D), ATCC VR-878 (G) and ATCC VR-901BD (LGV1) in order to calculate the DNA copy number, using NEBioCalculator (http://nebiocalculator.neb.com/#!/dsdnaamt) according to the following formulas:

$$ \mathrm{moles}\ \mathrm{dsDNA}\ \left(\mathrm{mol}\right)=\mathrm{mass}\ \mathrm{of}\ \mathrm{dsDNA}\ \left(\mathrm{g}\right)/\left(\left(\mathrm{length}\ \mathrm{of}\ \mathrm{dsDNA}\ \left(\mathrm{bp}\right)\times \kern0.37em 617.96\;\mathrm{g}/\mathrm{mol}\right)+\kern0.37em 36.04\;\mathrm{g}/\mathrm{mol}\right) $$

$$ \mathrm{moles}\ \mathrm{of}\ \mathrm{dsDNA}\ \mathrm{ends}=\mathrm{moles}\ \mathrm{dsDNA}\ \left(\mathrm{mol}\right)\times \kern0.37em 2 $$

$$ \mathrm{DNA}\ \mathrm{copy}\ \mathrm{number}=\mathrm{moles}\ \mathrm{of}\ \mathrm{dsDNA}\times \kern0.37em 6.022\mathrm{e}23\ \mathrm{molecules}/\mathrm{mol} $$

Droplet Digital PCR (ddPCR) data of *C. trachomatis* DNA copy number was available from ATCC for strain ATCC VR-901BD. The actual DNA copy number (by ddPCR) was used for estimation of the actual copy number in strains ATCC VR-885, ATCC VR-878. The stock DNA was serially diluted in PCR GRADE WATER according to Table 4 and 8 μl of each dilution was used for performing MLST as indicated. The amplification quality was scored according to our Good/Moderate/Bad scheme and only Good score was considered as successful amplification. In a similar experiment 912, 91.2 DNA copy number of ATCC VR-885, VR-901BD were spiked into 400 μl of negative urine (as determined by GeneXpert). The DNA was purified using MagNa pure compact and the MLST was performed as indicated with a successful amplification (not presented).

### Comparative analysis

Thirty-two urine samples that were found positive for *C.trachomatis* by GeneXpert test were chosen for the comparative study. For details about each sample see Table S8 in the supplementary section. These samples are part of the 81 urine sample that were used in this study (Table S9 in the supplementary section). The samples represent various DNA qualities, DNA concentration, as well as various genovars and STs. Each sample was analyzed once with the method presented in this study, or by Bom’s MLST primers scheme [28].

## Supplementary Information

**Additional file 1.**

## Data Availability

The datasets generated during the current study are available in the NCBI GenBank repository, under accession numbers, *ompA*: MW258775 - MW258830; CT046 (*hctB*): MW309910 - MW309962; CT058: MW309963 - MW310018; CT144: MW310075 - MW310130; CT172: MW310019 - MW310074; CT682 (*pbpB*): MW310131 - MW310186.

## Abbreviation

MLST: Multilocus Sequence Typing
